# Environmental Risk Factors Implicated in Liver Disease: A Mini-Review

**DOI:** 10.3389/fpubh.2021.683719

**Published:** 2021-06-24

**Authors:** Rajesh Melaram

**Affiliations:** School of Health Sciences, Walden University, Minneapolis, MN, United States

**Keywords:** aflatoxin, environmental, hepatocellular, carcinoma, liver disease, microcystin, pesticide

## Abstract

Liver disease is a global health issue, resulting in about two million deaths per year. It encompasses a wide spectrum of varied or unknown etiologies, ranging from lifestyle choices to pre-existing comorbidities. In recent decades, exposure to environmental toxins and subsequent liver health outcomes have captured public interest, due to the extensive application of pesticides, consumption of aflatoxin contaminated foodstuff, and cyanobacterial harmful algae blooms in endemic regions of liver disease. Hepatocellular carcinoma is a serious and debilitating condition of the liver, characterized by abdominal pain and unexplained weight loss. Established risk factors for hepatocellular carcinoma include alcohol consumption, cigarette smoking, and viral infections of hepatitis B and C. However, mounting evidence suggests that environmental toxins may represent an important contributing factor in hepatocellular carcinoma development. This mini-review synthesizes epidemiological investigations, providing evidence for environmental toxins as one potential risk factor for liver disease.

## Introduction

Liver disease is a global health problem causing approximately two million deaths annually, owing to cirrhosis, hepatocellular carcinoma, and viral hepatitis. One million liver disease deaths occur from cirrhosis complications, while another million results from viral hepatitis and hepatocellular carcinoma (1). Despite strides in antiviral and vaccine developments, liver disease represents a significant burden to society and continues to worsen as life expectancy grows with sedentary lifestyles and overnutrition (2). The largest burden of liver disease rests in Europe, with cirrhosis and liver cancer increasing throughout most European countries. Liver disease epidemiology varies across Europe due to the prevalence of modifiable risk factors, including heavy alcohol consumption, obesity, and viral hepatitis (3). In the United States of America (USA), chronic liver disease and cirrhosis are responsible for >44,000 deaths each year, regardless of underestimates in liver deaths (4). Alcohol-related liver disease, chronic hepatitis B virus, hepatitis C virus, and non-alcoholic fatty liver disease are common etiologies of chronic liver disease and cirrhosis (5). Global estimates on chronic liver disease and cirrhosis indicate non-alcoholic fatty liver disease (60%) as the most common etiology, followed by hepatitis B virus (29%), hepatitis C virus (9%), and alcoholic-related liver disease (2%) (6). Moreover, China, a developing country where >20% of population is affected by countless liver diseases, is experiencing an upsurge in liver disease burden (2). Important causes of liver morbidity and mortality in China include alcohol-related liver disease, drug-induced liver injury, hepatitis B and C virus infections, liver cancer, liver cirrhosis, and non-alcoholic fatty liver disease. Although heavy alcohol consumption, viral hepatitis, and non-alcoholic liver disease are major risk factors for liver disease worldwide (7), recent evidence indicates that environmental toxins (organochlorine pesticides, aflatoxins, microcystins) contribute to liver disease.

Pesticides, chemicals used to manage and treat pests, have been linked to human cancers (8). The agricultural and horticultural industries widely employ pesticides, and human exposure primarily occurs via diet (9, 10). Some factors prevent degradation among household pesticides, such as lack of moisture, microorganisms, and sunlight (10, 11), which may facilitate human exposure through dermal contact and ingestion (12). Experimental studies have demonstrated that organochlorine exposure, specifically dichlorodiphenyltrichloroethane (DDT) and dichlorodiphenyldichloroethylene (DDE), results in liver tumors and hepatocellular carcinoma development in rodents (9, 13–15). Yet, epidemiological studies examining pesticide exposure and human hepatocellular carcinoma have produced mixed results, particularly in the USA. For example, one study determined that farmers were at an increased risk for HCC compared to non-farmers (16), while three other studies indicated a non-significant increased risk for HCC among farmers (17–19).

Aside from pesticides, aflatoxins have shown to increase the risk of liver disease. Aflatoxins comprise a group of mycotoxins produced by the toxigenic species, *Aspergillus flavus* and *Aspergillus parasiticus*. Aflatoxin B1, a contaminant of dietary staples (groundnuts, maize, rice, and sorghum) in tropical and subtropical regions (20), is the main aflatoxin of concern to humans. High temperatures and humidity, in conjunction with plant moisture content, are factors of fungal growth and toxin production (21). Globally, risk of aflatoxin exposure is estimated to affect 4.5 to 5.5 billion people (22). Southeast Asia, sub-Saharan Africa, and some parts of South America experience the highest risk of exposure to aflatoxins. Aflatoxin B1 exposure may be responsible for approximately between 25,200 and 155,000 HCC cases worldwide, and an estimated 40% live in sub-Saharan Africa (22).

Liver disease incidence is reportedly high in places enduring cyanobacterial harmful algae blooms (23–26). These phenomena result from photosynthetic cyanobacteria multiplying within freshwater systems given favorable environmental factors, such as light intensity, nutrients, pH, short-wavelength radiations, and temperature (27–29). Cyanobacterial harmful algae blooms can release several cyanotoxins into surrounding waters, including anatoxins, cylindrospermopsins, microcystins, nodularins, and saxitoxins (30). However, the microcystins constitute an important and prevalent cyanotoxin in surface waters globally, posing environmental and health hazards (31). Many cyanobacterial genera synthesize microcystins, including colonial *Microcystis* spp. and filamentous *Anabaena* spp., *Anabaenopsis* spp., *Aphanizomenon, Nostoc*, and *Planktothrix*/*Oscillatoria* (32). Their mode of action entails the inhibition of protein phosphatases 1A and 2B in hepatocytes, where they accumulate to induce liver damage (33, 34). Acute poisoning can interfere with liver function, promoting hemorrhage formation, and ultimately, hemorrhagic shock (35, 36). Also, liver toxicity caused by microcystin has shown to induce apoptosis, cytoskeletal disruption, DNA damage, inflammation, necrosis, and oxidative stress (37, 38).

Ingestion of contaminated drinking water is a frequent source of human exposure, although microcystins can taint aquatic organisms for consumption, harbor waters where dermal contact occurs, and drift as aerosol sprays during recreational activity (39). Acute exposure causes dermatitis, fever, headache, increased liver enzyme activity, and stomachache (40). Signs and symptoms of chronic exposure are less clear, regardless of a possible connection with an increased liver disease risk. Several epidemiological studies identified potential linkages between microcystin exposure and liver disease (23–26, 40). Fatal intoxications rarely happen, as recounted in Brazil, where cyanotoxin exposure was considered a contributing factor for the death of hemodialysis patients (41).

This mini-review investigates the linkage between environmental toxin exposure and liver disease in endemic regions. Hepatocellular carcinoma, a major cause of primary liver disease, is examined in the context of human environmental exposure. Therefore, we review and analyze pertinent epidemiological data on hepatocellular carcinoma, supporting environmental toxins as an emergent risk factor.

## Hepatocellular Carcinoma

Hepatocellular carcinoma (HCC), a predominant form of primary liver cancer, is the third leading cause of cancer mortality worldwide. In the USA, HCC incidence has increased in recent decades and is expected to rise in the next 20 years as more individuals are diagnosed with hepatitis C virus and non-alcoholic steatohepatitis (42). From 2000 to 2012, adjusted incident rates for HCC increased by an average annual percentage change of 4.5% (95% CI 4.3–4.7) (43). The main risk factors for HCC in the USA, starting with the greatest burden for HCC, include non-alcoholic fatty liver disease, alcoholic liver disease, and hepatitis C and B viruses (44). In China, HCC is the major histological type of liver cancer, comprising 83.9–92.3% of liver cancer cases (45). Approximately 19% of the world's population resides in China, and liver cancer incidence is higher compared to other nations (46).

### Hepatitis B and C Viruses

Primary liver cancer is a widespread disease of different varieties, with HCC accounting for about 75–85% of primary liver cancers (47). Chronic infection with hepatitis B virus (HBV) or hepatitis C virus (HCV) is a major risk factor for HCC, and HBV is the major risk factor in high incident areas of HCC (48). For instance, approximately 55% of HCC cases worldwide are due to chronic HBV infection, whereas an estimated 89% of HCC cases occur in regions endemic or hyperendemic to HBV (49, 50). In the USA, HCV is a major risk factor for HCC, where roughly 2% of the population has the disease (48). HCC in China is largely attributed to chronic hepatitis B infection, which is acquired early in life. Behavior intervention and vaccination have shown to reduce liver cancer incidence in endemic China (51). Universally, the availability of the HBV vaccine has resulted in fewer infections and resultant HCC cases. Similarly, the development of an HCV vaccine is key to a global control and elimination of HCV (52, 53).

### Alcohol Consumption and Cigarette Smoking

Alcohol consumption is a recognized risk factor for liver cirrhosis and HCC. In developed nations, such as the USA and Europe, alcohol consumption is frequent and regarded as one common etiology of HCC. Depending on the country and geographic area, the ratio of alcohol abuse to all HCC etiologies varies, and roughly 15–30% of HCC is attributed to alcohol abuse (54). Multiple studies demonstrated an association between a high alcohol consumption and an increased HCC risk. For example, as reviewed in (55), an alcohol intake of >60–100 g/day increases the risk for HCC, while an alcohol intake of >600,000 ml over a person's lifespan significantly increases their risk for HCC. Furthermore, the dose-effect relationship between alcohol consumption and HCC incidence has been studied in males and females, individually, with chronic HBV infection. Individuals with a high alcohol consumption and HBV (OR 48.6 CI 95% 24.1–98.0), or HCV (OR 109 CI 95% 50.9–233.0), had an increased risk for HCC than individuals with a low alcohol consumption and without chronic viral infection (56).

The role of cigarette smoking has been examined in relation to HCC mortality, but its effects remain uncertain. Cigarette smoking is known to induce toxicity and serves as initiators and promoters of various cancers (57). A large cohort study in China demonstrated an association between cigarette smoking and HCC mortality in females, accounting for alcohol consumption and dietary habits (58). Similarly, a large cohort study in Japan determined that cigarette smoking (past and present) was an important risk factor for HCC mortality. The study conducted a univariate analysis, meaning potential confounders and interactive effects potentially altered the observed association (59).

A meta-analysis of 38 cohort and 58 case-control studies explored the association of cigarette smoking with an increased risk in liver cancer development. The adjusted meta-analysis risk ratio for liver cancer among current smokers and former smokers was 1.51 (95% CI 1.37–1.67) and 1.12 (CI 95% 0.78–1.60), respectively. Irrespective of location, publication time, sample size, and study design, epidemiological studies within the meta-analysis concluded an increased risk for liver cancer among current smokers. The number of cigarettes smoked per day also positively correlated with liver cancer (60). In Hawaii, a retrospective study concluded a non-significant association between smoking and HCC survival. However, significant associations were reported for alcohol consumption and hepatitis B. Using a multivariable model, another retrospective study confirmed cigarette smoking as a non-significant independent predictor of HCC mortality. Unlike previous studies, the study evaluated the interaction between alcohol consumption and cigarette smoking, which was statistically significant (*p* = 0.02) (61).

### Pesticides

Pesticides are chemical agents used to control animals, plants, and microorganisms. Pesticide use in agricultural settings, commerce, individual households, and public health is large, increasing the likelihood of human exposures. Diet is a primary source of exposure in the USA as pesticides are routinely used in agriculture and horticulture. Alternative sources of exposure include occupation and residential proximity to agricultural pesticide applications (9, 10). It has been hypothesized that pesticide exposure contributes to hepatic carcinogenesis via genotoxic and immunotoxic mechanisms, in addition to hormonal action and tumor promotion (9). Additionally, epidemiological studies in the USA and China support an association between pesticide exposure and HCC risk. Case-control studies from China documented significant increased risks for HCC and organochlorine pesticides (62–64). Studies in the USA reported inconclusive results on pesticide exposure and HCC risk. Three studies indicated a non-significant increased risk for HCC among farmers (17–19), whereas one study revealed a higher increased risk of HCC among farmers compared to non-farmers (16). A more recent case-control study using a geographic information system demonstrated a significant association between organochlorine pesticides and increased HCC risk in males (OR 2.76 95% CI 1.58–4,82), but not in females (OR 0.83 95% CI 0.35–1.93) (65). Collectively, epidemiological studies in China and the USA provide evidence that pesticide exposure in agriculturally concentrated areas increases HCC risk in farmers.

### Aflatoxins

Aflatoxins are mycotoxins produced by *Aspergillus flavus* and *Aspergillus parasiticus*. These fungi thrive in hot and humid environments, which is promising for mycotoxin production. Aflatoxin B1 (AFB_1_), a potent mycotoxin of *Aspergillus* spp., is carcinogenic to experimental animals (66). Following its ingestion, AFB_1_ is metabolized by the hepatic cytochrome P-450-dependent monooxygenase system to a potent AFB_1_-8,9 oxide (67). This metabolite forms DNA and protein adducts through covalent interactions, for example, AFB_1_-guanine and AFB_1_-albumin (68). Research on aflatoxin metabolism and toxicology has resulted in the development of exposure biomarkers to assess its role in hepatocarcinogenicity. Many of these biomarkers involve aflatoxin metabolites in urine, DNA and protein adducts in blood and tissue, and excreted urinary guanine adduct (48).

AFB_1_ frequently contaminates food items, including corn, legumes, and peanuts, and human exposure results from consuming such products (69). Moreover, aflatoxins have been implicated in HCC incidence, predominantly in sub-Saharan Africa, Southeast Asia, and China. These regions include developing countries with tropical and subtropical climates, which favor *Aspergillus* spp. growth, thus increasing aflatoxin exposure (22). Many African and Asian diets involve staples of groundnuts and maize, two crops prone to aflatoxin infection. Many epidemiological studies documented associations between AFB_1_ exposure biomarkers and HCC risk, and data show that dietary AFB_1_ exposure may explain the high incidence of HCC in sub-Saharan Africa (70).

HBV is problematic in developing countries where chronic aflatoxin exposure occurs, and both risk factors affect rural populations more significantly than urban populations. The disparity may result from fewer dietary options in rural areas compared to urban areas. In rural areas, HBV prevalence is generally high, and viral infection is greater among males than females (71). AFB_1_ exposure and HBV infection often co-occur, making it difficult to measure individual exposure to AFB_1_, considering variations in toxin concentrations of food samples. Consequently, published studies report inconsistent findings concerning dietary AFB_1_ exposure and HCC risk. Collecting AFB_1_ biomarkers before a diagnosis of HBV or HCV may benefit prospective studies, allowing researchers to examine the interaction between AFB_1_ exposure and HBV or HCV infections relative to HCC development.

### Microcystins

The microcystins (MCs) represent a large group of cyanotoxins in the environment. These bioactive metabolites are low in molecular weight and can reach micromolar concentrations in bloom-infested waters. Various environmental parameters influence MC production within freshwater ecosystems, including pH, nitrogen and phosphorus, stochiometric ratio of available nitrogen to phosphorus, and water temperature (72). MCs contain a unique molecular substructure, 3-amino-9-methoxy-2,6,8-trimethyl-10-phenyl-deca-4,6-dienoic acid (Adda). Two variable sites (2 and 4) within the heptapeptide differentiates individual congeners (73). With over 250 congeners identified to date, MC-LR (leucine, arginine) is the most studied and toxic variant of MCs (74). Oral consumption of contaminated drinking water is the primary route of exposure to MCs. Uptake is dependent on a bile acid transporter facilitated by organic anion-transporting proteins expressed in hepatocytes. Once in the liver, MCs inactivate protein phosphatases types 1 and 2A, triggering liver failure (33–35). Thus, MCs are classified as hepatotoxins, and human exposure is vastly a concern in areas with a high endemicity of liver cancer (Table 1).

**Table 1:** Summary of epidemiological investigations on microcystins and liver disease.

**Authors**	**Country**	**Design**	**Environmental toxin**	**Finding**
Ueno et al. (23)	China	Epidemiological survey	Microcystin	MC detection in drinking water sources correlates with a high PLC incidence
Fleming et al. (25)	USA	Pilot ecological study	Microcystin	Residential proximity to surface water drinking sources increases HCC risk
Chen et al. (75)	China	Longitudinal study	Microcystin	Concurrent detection of serum MCs and liver enzymes indicate hepatocellular damage in fishermen
Svirčev et al. (24)	Serbia	Descriptive epidemiological method	Microcystin	Significant and persistent blooms correlate with PLC mortality and incidence
Zheng et al. (26)	China	Case-control study	Microcystin	Serum MC detection in patients link to HCC risk

An epidemiological survey containing three trials evaluated MC exposure and PLC in endemic China (23). The first trial discovered blue-green algal hepatotoxins in ditch/pond samples (21%), ranging from 90 to 460 pg/ml. In a second trial, MC concentrations increased from June to September (62–296 pg/ml). The third trial revealed MCs in drinking water sources of ditches/ponds, rivers, and shallow wells. Most contamination occurred in river samples (32%), followed by ditch/pond samples (17%) and shallow well samples (4%). Results of the epidemiology survey supported blue-green algal hepatotoxins in drinking water as one potential risk factor for China's high PLC incidence.

Previous findings on MC exposure and PLC incidence prompted hepatotoxin analysis in human serum. Daily chronic exposure to MCs and ensuing health effects were studied in fishermen at Lake Chaohu (75). Liquid chromatography-mass spectrometry detected MCs in 35 samples, averaging 0.389 ng/mL. Compared to the World Health Organization's tolerable daily intake for lifetime daily exposure of 2.0–3.0 μg/person, a range of 2.2–3.9 μg MC-LR equivalents was estimated as the daily intake of MCs. Multivariable analyses characterized a positive relationship between serum liver enzymes and MCs, indicating hepatocellular damage in fisherman from chronic exposure to MCs. The study demonstrated a biochemical biomarker for MC exposure via serum liver enzyme measurement. Several years later, a positive correlation was discovered for serum MC-LR and HCC risk in southwest China (7). Statistical analyses were controlled for the established risk factors, including HBV, alcohol, and aflatoxin. The odds ratio for HCC risk increased by 2.3 (95% CI 1.5–5.5) as an elevated serum MC-LR was detected in patients. Binary logistic regression determined a positive interaction with alcohol (synergism index = 4.0 95% CI 1.7–9.5) and HBV (synergism index = 3.0 95% CI 2.0–4.5), but a negative interaction with aflatoxin (synergism index = 0.4 95% CI 0.3–0.7). The results confirmed serum MC-LR as an independent risk factor for HCC risk.

Besides China, liver cancer is a malignant disease in Central Serbia and the southeastern region of the USA. Two investigation periods (1980–1990 and 2000–2002) were conducted on cyanotoxin exposure and PLC mortality and incidence in Central Serbia (24). Heavy cyanobacterial blooms occurred in regions with a high PLC mortality (11.6 for 1980–1990) and PLC incidence (34.7 for 2000–2002). A drinking water reservoir contained an elevated MC-LR concentration of 650 μg/L compared to 2.5 μg/L from tap water. Based on descriptive epidemiological data, persistent and significant blooms correlated with PLC morality and incidence. The study lacked statistical models, which could strengthen the argument that hepatotoxins in drinking water reservoirs correlate with PLC mortality and incidence. Following the detection of MCs in surface water drinking sources, a pilot ecological study in Florida assessed the proximity to a surface water treatment plant and HCC risk (25). Environmental databases connected HCC cancers diagnosed between 1981 and 1998. Residents who lived within service zones had an increased risk for HCC than residents who lived in neighboring zones. Study results limited to the population level as the design was ecological, a limitation of ecological studies. Conversely, exposure assessments can identify toxicants and their effects to determine whether a causal relationship exists between environmental exposure and adverse health outcome. The validity and variability of MC biomarkers, however, may present a challenge in defining an exposure measurement.

## Conclusion

Liver disease is a multifactorial disease of identified factors, including alcohol consumption, cigarette smoking, and hepatitis B and C virus infections. Limited epidemiological investigations speculate the association between environmental toxin exposure and hepatocellular carcinoma development in endemic regions. Hepatocellular carcinoma, a regular cause of cancer death across the world, is continuing to grow in developing and developed nations. Herein, environmental exposure constitutes a public health hazard due to the increased usage of organochlorine pesticides in occupational settings, dietary staples perpetually contaminated with aflatoxin B1, and continued presence of hepatotoxic microcystins in drinking water sources. This mini-review examined multiple epidemiological studies on hepatocellular carcinoma in an effort to illuminate human exposure to environmental toxins as one emergent risk factor for liver disease. We found that most epidemiological data support the potential association between environmental toxins and hepatocellular cancer in the developing world. Notably, microcystin pollution in drinking water sources appears to greatly influence liver cancer, a problem worth researching due to changes in climate and personal lifestyle behaviors. Findings can be used to assist health and medical professionals in all levels of prevention, including the diagnosis and treatment of liver disease patients.

## Author Contributions

RM wrote and edited all components of the manuscript.

## Conflict of Interest

The author declares that the research was conducted in the absence of any commercial or financial relationships that could be construed as a potential conflict of interest.
